# Association between frailty and cognitive function in hospitalized older adults: a moderated serial mediation analysis involving functional status, depression, and social support

**DOI:** 10.3389/fpubh.2026.1895486

**Published:** 2026-09-28

**Authors:** Jingrong Dai, Jing Huang, Fang Liu, Yangfan Hu, Hong Huang, Yan Li, Tingjun Wang

**Affiliations:** 1 Department of General Practice, National Regional Medical Center, Binhai Campus of the First Affiliated Hospital, Fujian Medical University, Fuzhou, China; 2 Department of General Practice, The First Affiliated Hospital, Fujian Medical University, Fuzhou, China; 3 Department of General Practice, The Affiliated Changsha Hospital of Xiangya School of Medicine, Central South University, Changsha, China; 4 Department of Geriatrics, Yunnan First People's Hospital, Kunming, China; 5 General Practitioner Branch of Fujian Medical Doctor Association, Fuzhou, China

**Keywords:** activities of daily living, cognitive function, depression, frailty, social support

## Abstract

**Objective:**

This study aimed to examine whether impaired basic activities of daily living (BADL) and depression accounted for the association between frailty and cognitive function in hospitalized older adults, and whether social support moderated these relationships.

**Methods:**

A cross-sectional design was adopted in this study, including hospitalized older adults aged 60 years and above. Data were collected using a digital comprehensive geriatric assessment system, including mini mental state examination (MMSE), frailty scale, BADL scale, geriatric depression scale (GDS), and social support rating scale (SSRS). Hierarchical regression, serial mediation, and moderated mediation analyses were performed using PROCESS macro (Model 92) with 5,000 bootstrap resamples to examine potential statistical pathways from frailty to cognitive function and the moderating role of social support.

**Results:**

Among 1,611 hospitalized older adults, frailty was statistically associated with lower MMSE scores. After adjustment for demographic, socioeconomic, and lifestyle-related variables, the direct association was not significant (*p* > 0.05), while the total indirect association was significant, statistically accounting for 88.8% of the observed total association within the specified model. Mediation analysis showed that the indirect association through the pathway of depressive symptoms was the largest, followed by the pathway through BADL and the sequential pathway via BADL and depressive symptoms. In the moderated serial mediation model, social support statistically moderated the link of frailty with BADL (*B* = 0.120, 95% CI 0.056–0.185, *p* < 0.001) as well as the link of GDS with MMSE (*B* = 0.014, 95% CI 0.005–0.023, *p* = 0.004). Further analysis indicated that as the level of social support increased, the indirect association through the pathways involving depressive symptoms and the sequential pathway gradually decreased. The moderation of social support was statistically significant for the pathways linking frailty with cognitive function through GDS (index = 0.134, 95% CI 0.039–0.227) and through BADL and GDS (index = 0.051, 95% CI 0.025–0.078).

**Conclusion:**

The relationship between frailty and cognitive function was mainly characterized by indirect associations involving BADL impairment and depression, with the depression pathway contributing the most statistically. Social support moderated specific associations within this statistical framework. These findings suggest that functional, psychological, and social factors should be considered together in efforts to maintain cognitive health and promote healthy aging among older adults.

## 1 Introduction

With the acceleration of global population aging, the number of people with cognitive impairment and dementia is increasing rapidly, posing a major public health challenge (1). Studies indicate that cognitive impairment is often found to coexist with multiple geriatric syndromes and is influenced by various factors, including physical vulnerability, functional decline, and psychosocial factors (2–5). Among these, frailty, as a syndrome reflecting reduced functional reserve across multiple systems, is closely related to cognitive impairment (6). Frailty in older adults is often accompanied by functional limitations and psychological burdens such as depressive symptoms, both of which have been associated with poorer cognitive performance (7, 8). However, previous studies have mostly focused on the association between two factors, and the potential relationships among frailty, functional status, psychological factors, and cognitive function remain insufficiently understood.

Studies have suggested that the association between frailty and cognitive function may involve multiple intermediate factors (9, 10). Previous studies have shown that functional impairment is closely associated with depression, and depression is significantly related to cognitive impairment, suggesting that functional status and psychological factors play important roles in the association between frailty and cognitive decline (11, 12). However, it remains unclear whether these factors are independently associated with cognitive function or are involved in a more complex relationship, and the structure of these potential pathways requires further investigation. In addition, social support has been regarded as an important psychosocial factor associated with cognitive function in older adults (13, 14). However, previous studies have rarely integrated frailty, functional impairment, depressive symptoms, and social support within a unified statistical framework, and whether social support modifies specific relationships within this framework remains insufficiently explored.

Therefore, this study aimed to construct a moderated serial mediation framework to explore the associations among frailty, functional status, depression, and cognitive function, and to examine whether social support moderated specific relationships within this framework.

## 2 Methods

### 2.1 Study design and subjects

This was a cross-sectional study based on data derived from the Comprehensive Geriatric Assessment (CGA) system, a digital platform developed by the Department of Geriatrics, the First People’s Hospital of Yunnan Province. The system was designed to facilitate the electronic administration of standardized geriatric assessment instruments and enable systematic data collection in routine clinical practice. From May 2021 to December 2024, a total of 1,800 patients aged 60 years or older who underwent CGA assessment during hospitalization were identified from the inpatient database of the Department of Geriatrics, the First People’s Hospital of Yunnan Province. According to the predefined exclusion criteria, 189 subjects were excluded, including duplicate records (*n* = 16), confirmed diagnosis of Alzheimer’s disease (*n* = 35), terminal illness with a life expectancy of less than 6 months (*n* = 12), and missing data for key variables (*n* = 126), including demographic variables and CGA-related assessment variables. Participants with missing data for key variables were excluded using a complete-case approach because these variables were required for the mediation analyses. The distribution of missing data was examined before analysis. A total of 1,611 patients were ultimately included in the analysis (Figure 1). The procedures of the study were in accordance with the Declaration of Helsinki, and written informed consent was obtained from each participant involved. This study was approved by the Medical Ethics Committee of the First People’s Hospital of Yunnan Province (Approval No. KHLL2021-KY079).

**Figure 1:**
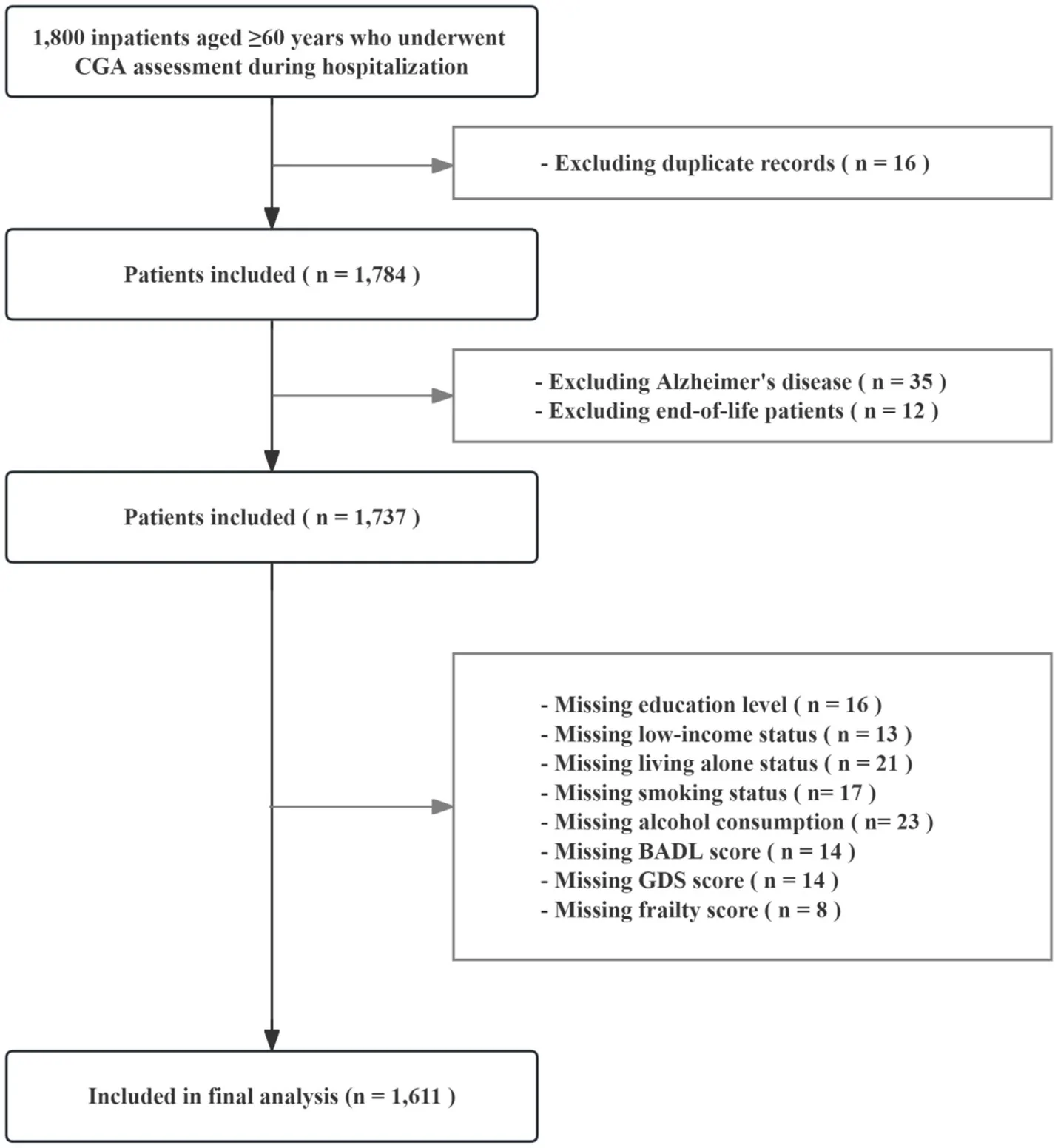
Flow chart of this study. BADL, basic activities of daily living; GDS, geriatric depression scale.

### 2.2 Conceptual framework and hypotheses

A theoretical framework incorporating mediating and moderating associations was constructed in this study to explore the relationship between frailty and cognitive function in hospitalized older adults (Figure 2). Within this framework, basic activities of daily living (BADL) and depression were included as potential mediators to examine whether these factors statistically accounted for part of the association between frailty and cognitive function. Previous evidence suggests that frailty is associated with functional decline, and impaired functional status is statistically associated with depressive symptoms, possibly through reduced independence and social participation (7, 11, 12). Therefore, BADL impairment and depression were hypothesized to represent sequentially organized components within the statistical framework linking frailty and cognitive function. Social support was introduced as a moderating variable because psychosocial resources may modify specific associations among functional status, depressive symptoms, and cognitive function. As an important psychosocial resource, social support may attenuate the adverse associations of functional impairment and depressive symptoms with cognitive function (13, 14). Therefore, a moderated serial mediation framework was applied to explore whether social support modified specific associations within the proposed statistical pathway. This approach was selected because it allows simultaneous examination of whether social support modifies different associations within a serial mediation framework. Accordingly, the following hypotheses were proposed: (1) frailty would be associated with cognitive function; (2) BADL impairment and depressive symptoms would statistically account for part of this association, including a potential sequential association from BADL impairment to depressive symptoms; and (3) social support would moderate specific associations within this statistical framework.

**Figure 2:**
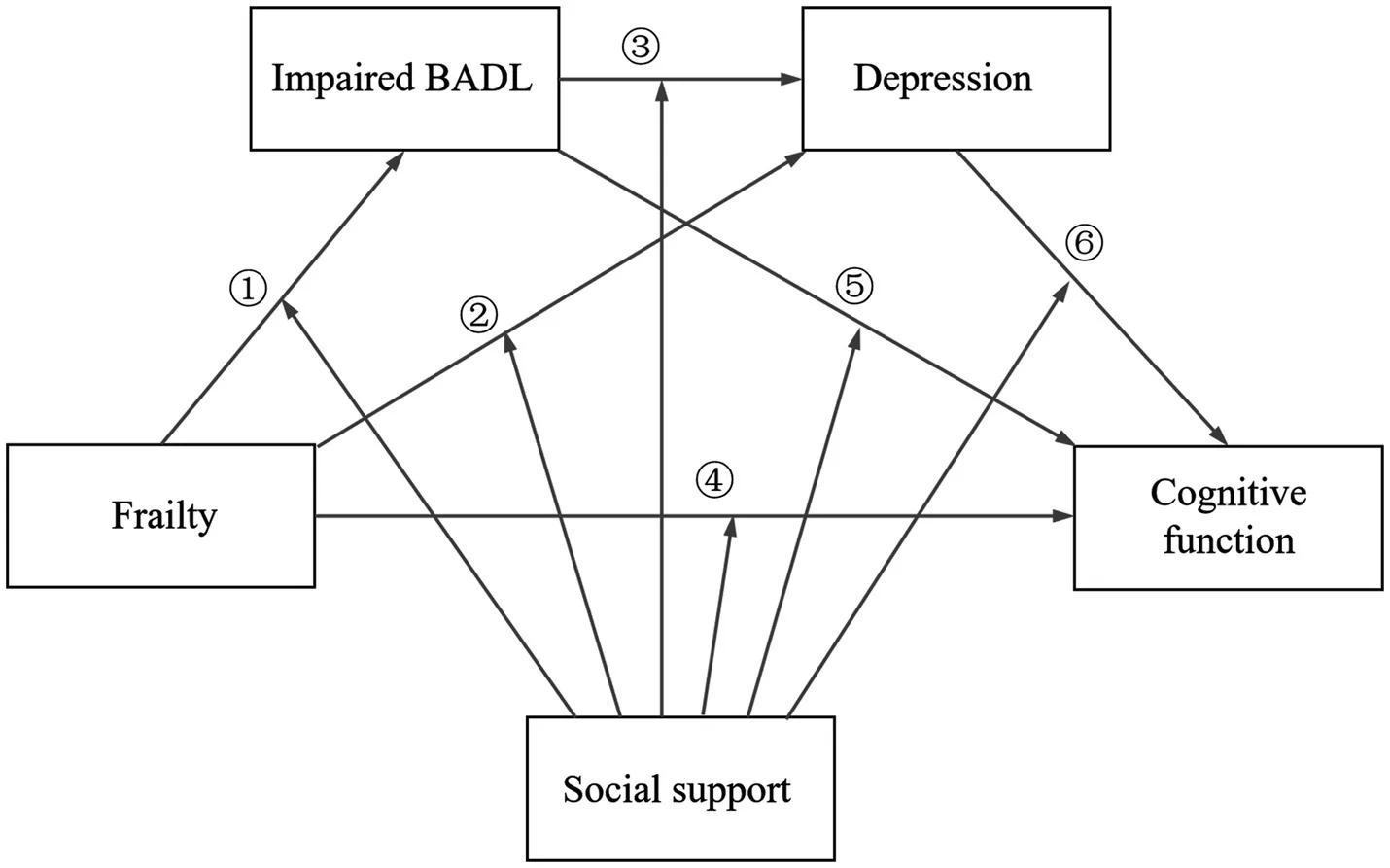
Conceptual moderated serial mediation model and hypothesized moderating pathways of social support. The numbered pathways represent hypothesized interaction terms involving social support examined using PROCESS Model 92: ① Frailty × Social support → Impaired BADL; ② Frailty × Social support → Depression; ③ Impaired BADL × Social support → Depression; ④ Frailty × Social support → Cognitive function; ⑤ Impaired BADL × Social support → Cognitive function; ⑥ Depression × Social support → Cognitive function. BADL, basic activities of daily living.

### 2.3 Assessment procedure and quality control

The investigators involved in the assessment were all geriatric medicine experts proficient in CGA techniques. They had all received standardized training in the CGA software and received standardized training in CGA assessment procedures. This assessment was conducted through face-to-face interviews. Demographic, socioeconomic, and lifestyle-related data, including sex, age, marital status, education level, living arrangement, low-income status, smoking, and alcohol consumption, were collected. Marital status was categorized as married with a living spouse, widowed, divorced, or unmarried. Education level was categorized into five ordered groups: illiterate, primary school, junior high school, senior high school, and college or above. Living arrangement was classified as living alone or not living alone. Low income status was defined according to whether the household received local minimum living allowance. Smoking status was classified as never smoking, former smoking defined as having stopped at least 6 months ago, or current smoking defined as smoking within the past 6 months. Drinking status was classified as never drinking, former drinking defined as having stopped at least 6 months ago, or current drinking defined as drinking within the past 6 months.

Cognitive function was assessed using the 30-point mini-mental state examination (MMSE), evaluating orientation, memory, attention, language, and visuospatial ability, and higher scores indicated better performance (15). For descriptive analyses, participants were classified as normal cognition or possible cognitive impairment using education-adjusted Chinese norm cutoffs: ≤17 for illiterate, ≤20 for primary school, and ≤24 for middle school or higher (16). Frailty was assessed using the 5-item FRAIL scale, evaluating fatigue, resistance, ambulation, illness burden, and weight loss. Each item was scored dichotomously (yes = 1, no = 0), yielding a total score of 0–5, with higher scores indicating increased frailty severity (17). BADL were assessed using a standardized scale evaluating independence in six fundamental self-care tasks: bathing, dressing, toileting, transferring, continence, and feeding. Each item was scored based on assistance required and total scores ranged from 0 to 100, with higher scores indicating greater functional independence and lower scores reflecting greater caregiver dependence (18). Depression was assessed using the 15-item geriatric depression scale (GDS), a brief yes/no screening tool for older adults, and mood, activity level, and outlook over the past week were evaluated. Scores ranged from 0 to 15, with higher scores indicating greater symptom severity (19). Social support was assessed using the Social Support Rating Scale (SSRS), a validated 10-item instrument covering objective support, subjective support, and support utilization. For further analysis, social support was categorized into low (≤−1 SD), medium (−1SD to +1SD), and high (≥+1 SD) levels. Total scores ranged from 0 to 40, with higher scores indicating greater social support (20).

### 2.4 Statistical analysis

All statistical analyses were performed using SPSS version 26.0 (IBM Corp., Armonk, NY, USA). Continuous variables were expressed as mean ± standard deviation, and comparisons between two groups were performed using independent-samples *t* tests. Categorical variables were presented as frequencies with percentages and compared using the chi-square test. Spearman correlation analysis was used to examine correlations among frailty, BADL, GDS, SSRS, and MMSE scores. After adjusting for age, sex, education level, marital status, low-income status, and living arrangement, hierarchical linear regression analysis was performed to examine the associations of frailty and social support with cognitive function as measured by the MMSE. Changes in explained variance (Δ*R*^2^) were calculated for each step. Model assumptions, including linearity, normality, and homoscedasticity of residuals, were assessed using residual diagnostic plots. Influential observations were evaluated using Cook’s distance. Multicollinearity among independent variables was assessed using variance inflation factors (VIFs) and tolerance values.

Moderating associations were examined by introducing interaction terms. Categorical variables were entered into regression models as dummy variables. Exploratory serial mediation and moderated serial mediation analyses were conducted using the PROCESS macro (Model 92) with 5,000 bootstrap resamples and 95% confidence intervals (CI). Model 92 was selected because it allows examination of whether a moderator modifies multiple associations within a serial mediation framework. Frailty was specified as the independent variable, MMSE as the dependent variable, BADL and GDS as sequential mediators, and SSRS as the moderator. Conditional indirect associations and the index of moderated mediation were calculated to characterize moderated statistical associations within the proposed framework. Because all variables were assessed at the same time point, mediation and moderated mediation analyses were interpreted as statistical associations within the proposed theoretical framework rather than evidence of temporal or causal relationships. In regression analyses, *β* represented standardized regression coefficients and *B* represented unstandardized regression coefficients. Completely standardized indirect effects were reported to facilitate comparison of indirect association magnitudes among variables measured on different scales. Boot SE represented the bootstrap standard error. A two-sided *p* value <0.05 was considered statistically significant.

## 3 Results

### 3.1 Participant characteristics

A total of 1,800 participants were initially identified, of whom 189 were excluded and 1,611 were included in the final analysis. No significant differences in available characteristics were observed between included and excluded participants (all *p* > 0.05; Supplementary Table S1). Table 1 presents the characteristics of participants divided by cognitive status. The mean age was 75.67 ± 8.57 years. Among them, 787 (48.8%) were male and 824 (51.2%) were female. Based on MMSE-defined cognitive status, 452 participants were classified as having normal cognition and 1,159 as having possible cognitive impairment according to education-adjusted MMSE cutoff values. Participants with possible cognitive impairment were older, more likely to be female, unmarried, less educated, of low-income status, and living alone compared to those with normal cognition (all *p* < 0.05). The participants with possible cognitive impairment showed significantly poorer BADL (86.83 ± 20.43 vs. 94.63 ± 13.19, *t* = −9.04, *p* < 0.001), higher scores of GDS (4.14 ± 3.43 vs. 2.23 ± 2.46, *t* = 12.37, *p* < 0.001) and higher frailty score (1.44 ± 1.29 vs. 1.01 ± 1.03, *t* = 6.99, *p* < 0.001), and lower scores of SSRS (34.47 ± 11.54 vs. 38.69 ± 12.67, *t* = −6.42, *p* < 0.001). No significant differences in smoking status and alcohol consumption were observed between the two groups (all *p* > 0.05).

**Table 1:** The characteristics of participants by the status of cognitive function (*n* = 1,611).

Variable	Category	Normal cognition (*n* = 452)	Possible cognitive impairment (*n* = 1,159)	*χ*^2^/*t*	*p* value
Sex (%)	Male	264 (58.4)	523 (45.1)	22.96	<0.001
	Female	188 (41.6)	636 (54.9)		
Age (years)	–	73.28 ± 8.19	76.60 ± 8.45	−7.17	<0.001
Marital status (%)	Married	407 (90.0)	916 (79.0)	26.85	<0.001
	Widowed/divorced/unmarried	45 (10.0)	243 (21.0)		
Education level (%)	Illiterate	78 (17.3)	433 (37.4)	100.17	<0.001
	Primary school	218 (48.2)	538 (46.4)		
	Junior high school	67 (14.8)	104 (9.0)		
	Senior high school	38 (8.4)	42 (3.6)		
	College or above	51 (11.3)	42 (3.6)		
Low-income status (%)	No	421 (93.1)	975 (84.1)	22.86	<0.001
	Yes	31 (6.9)	184 (15.9)		
Living alone (%)	No	403 (89.2)	908 (78.3)	24.32	<0.001
	Yes	49 (10.8)	251 (21.7)		
Smoking status (%)	Never	321 (71.0)	875 (75.5)	3.46	0.177
	Former	76 (16.8)	168 (14.5)		
	Current	55 (12.2)	116 (10.0)		
Alcohol consumption (%)	Never	366 (81.0)	986 (85.1)	4.53	0.104
	Former	48 (10.6)	104 (9.0)		
	Current	38 (8.4)	69 (6.0)		
BADL score	–	94.63 ± 13.19	86.83 ± 20.43	9.04	<0.001
GDS score	–	2.23 ± 2.46	4.14 ± 3.43	−12.37	<0.001
SSRS score	–	38.69 ± 12.67	34.47 ± 11.54	6.42	<0.001
Frailty score	–	1.01 ± 1.03	1.44 ± 1.29	−6.99	<0.001
MMSE score	–	28.941 ± 1.10	18.841 ± 5.56	38.294	<0.001

BADL, basic activities of daily living; GDS, geriatric depression scale; SSRS, social support rating scale; MMSE, mini-mental state examination.

### 3.2 Correlations among frailty, BADL, depression, social support, and cognitive function

As shown in Table 2, spearman correlation analysis indicated that MMSE score was negatively correlated with frailty score (*r_s_* = −0.216, *p* < 0.001) and GDS score (*r_s_* = −0.347, *p* < 0.001), and positively correlated with BADL score (*r_s_* = 0.257, *p* < 0.001) and SSRS score (*r_s_* = 0.301, *p* < 0.001). Frailty score was positively correlated with GDS score (*r_s_* = 0.396, *p* < 0.001) and negatively correlated with BADL *(r_s_* = −0.420, *p* < 0.001) and SSRS (*r_s_* = −0.229, *p* < 0.001).

**Table 2:** Spearman correlation matrix of the scores related to frailty, BADL, GDS, MMSE and SSRS (*n* = 1,611).

Variable	MMSE	Frailty	BADL	GDS	SSRS
MMSE	1				
Frailty	−0.216^*^	1			
BADL	0.257^*^	−0.420^*^	1		
GDS	−0.347^*^	0.396^*^	−0.319^*^	1	
SSRS	0.301^*^	−0.229^*^	0.208^*^	−0.399^*^	1

MMSE, mini-mental state examination; BADL, basic activities of daily living; GDS, geriatric depression scale; SSRS, social support rating scale, ^*^p < 0.05.

### 3.3 Associations of frailty and social support with cognitive function

Hierarchical regression analysis showed that, after adjusting for age, sex, education level, marital status, low-income status, and living arrangement, frailty score was negatively associated with MMSE score (*β* = −0.153, *p* < 0.001), whereas SSRS score showed a significant positive association with MMSE score (*β* = 0.142, *p* < 0.001). Frailty–SSRS interaction was also shown to be associated with MMSE score (*β* = 0.048, *p* = 0.035) (Table 3). The final model accounted for 17.6% of the variance in MMSE scores (adjusted *R*^2^ = 0.171), and the addition of the frailty-SSRS interaction term provided a significant incremental contribution (Δ*R*^2^ = 0.021, *p* < 0.001). In addition, multicollinearity diagnostics indicated that all variables included in the regression model had acceptable tolerance values (0.645–0.965) and VIF values (1.036–1.552), suggesting that no substantial multicollinearity was present.

**Table 3:** Hierarchical regression analysis of cognitive function (*n* = 1,611).

Variable	Model 1		Model 2		Model 3	
	*β*	*p*	*β*	*p*	*β*	*p*
Age	−0.179 (−0.231)	<0.001	−0.122 (−0.158)	<0.001	−0.123 (−0.159)	<0.001
Sex	−1.056 (−0.080)	0.001	−0.979 (−0.074)	0.002	−0.982 (−0.075)	0.002
Education level	1.239 (0.201)	<0.001	1.164 (0.189)	<0.001	1.165 (0.189)	<0.001
Marital status	−1.267 (−0.074)	0.003	−0.906 (−0.053)	0.029	−0.896 (−0.052)	0.031
Low-income status	−1.313 (−0.068)	0.004	−1.378 (−0.071)	0.002	−1.387 (−0.072)	0.002
Living alone	0.213 (0.007)	0.754	0.390 (0.013)	0.557	0.447 (0.015)	0.501
Frailty score (centered)	–	–	−0.815 (−0.153)	<0.001	−0.779 (−0.147)	<0.001
SSRS score (centered)	–	–	0.078 (0.142)	<0.001	0.079 (0.144)	<0.001
Frailty × SSRS	–	–	–	–	0.022 (0.048)	0.035
*R* ^2^	0.13		0.155		0.176	
Adjusted *R*^2^	0.126		0.151		0.171	
Δ*R*^2^	–		0.025		0.021	
*F*	39.832		47.347		20.716	
*p*	<0.001		<0.001		<0.001	

Model 1 included demographic variables of age, sex, education level, marital status, low-income status, and living arrangement. Model 2 further included frailty score and SSRS score. Model 3 additionally included the interaction term between frailty score and SSRS score. Frailty score and SSRS score being mean-centered prior to analysis. SSRS, social support rating scale. B represents unstandardized regression coefficient and *β* represents standardized regression coefficient. SSRS, social support rating scale.

### 3.4 Serial mediation analysis of the association between frailty and cognitive function

Serial mediation analysis indicated a significant total association between frailty score and MMSE score after adjustment for age, sex, education level, marital status, low-income status, and living arrangement (*B* = −0.898, 95% CI −1.153 to −0.642, *p* < 0.001). The direct association was not statistically significant (*B* = −0.101, 95% CI −0.387–0.185, *p* = 0.490). In contrast, the total indirect association was significant (*β =* −0.150, *B* = −0.797, 95% CI −0.982 to −0.619, *p* < 0.001). Significant specific indirect associations were observed across three pathways: via BADL alone (*β* = −0.053, *B* = −0.282, 95% CI −0.432 to −0.143, *p* = 0.002), via GDS alone (*β* = −0.075, *B* = −0.397, 95% CI −0.512 to −0.299, *p* < 0.001), and via the sequential statistical pathway from BADL to GDS (*β* = −0.022, *B* = −0.118, 95% CI −0.169 to −0.074, *p* < 0.001). These indirect associations statistically accounted for 31.4, 44.2, and 13.2% of the observed total association estimated within the specified model, respectively, with the pathway via GDS contributing the largest proportion (Table 4). The links with *B* (95%CI) in the pathway analysis involving frailty, BADL, GDS, and MMSE are presented in Figure 3. Similarly, the direct link of frailty with MMSE was not statistically significant (*p* > 0.05). The residual diagnostic analyses indicated that the assumptions of linearity, normality, and homoscedasticity were acceptable, and no influential observations were identified based on Cook’s distance.

**Table 4:** Serial mediation analysis for the indirect associations between frailty and cognitive function (*n* = 1,611).

Pathway	Completely standardized indirect coefficient (*β*)	Unstandardized coefficient (*B*)	Boot SE	95% Boot CI	*p* value	Proportion of total association (%)
Total association	–	−0.898	0.130	−1.153 to −0.642	<0.001	100
Direct association	–	−0.101	0.146	−0.387–0.185	0.490	11.2
Total indirect association	−0.150	−0.797	0.093	−0.982 to −0.619	<0.001	88.8
Frailty → BADL → MMSE	−0.053	−0.282	0.074	−0.432 to −0.143	0.002	31.4
Frailty → GDS → MMSE	−0.075	−0.397	0.055	−0.512 to −0.299	<0.001	44.2
Frailty → BADL → GDS → MMSE	−0.022	−0.118	0.024	−0.169 to −0.074	<0.001	13.2

All models were adjusted for age, sex, education level, marital status, low-income status, and living arrangement. Standardized coefficient (*β*) for indirect association was reported to facilitate comparison of indirect associations among variables measured on different scales. BADL, basic activities of daily living; MMSE, mini-mental state examination; GDS, geriatric depression scale.

**Figure 3:**
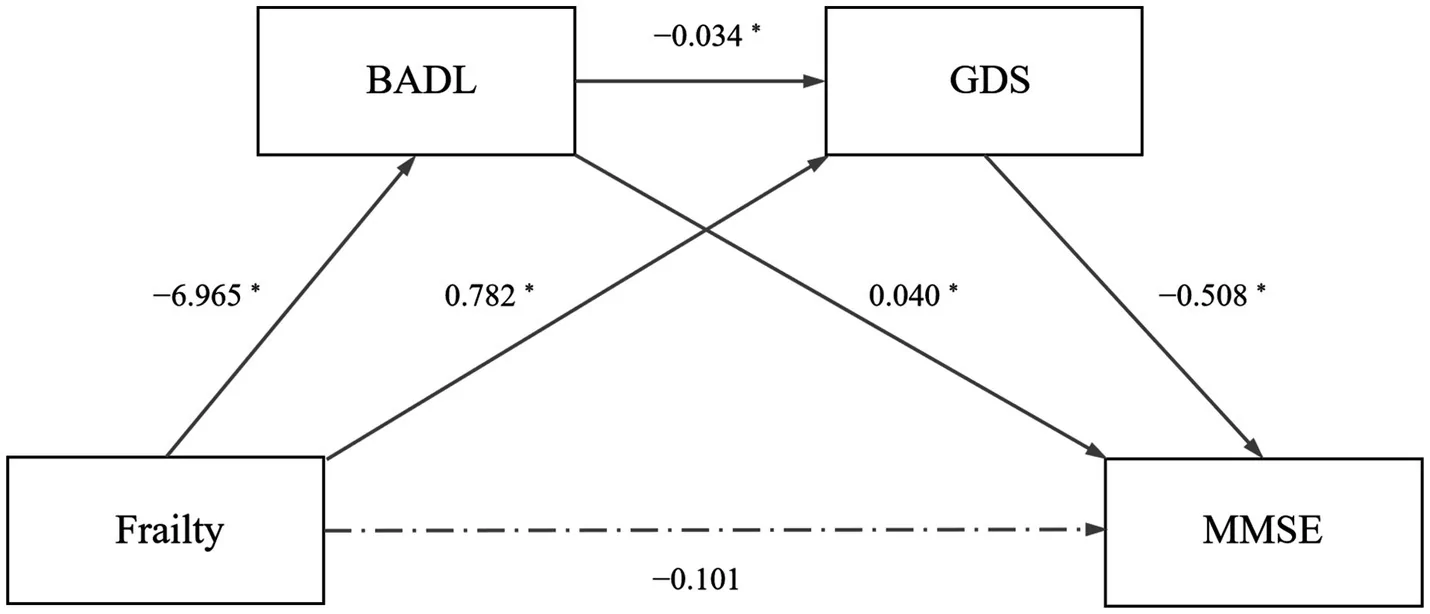
Statistical pathways involving frailty, BADL, GDS, and cognitive function identified in the serial mediation analysis. Solid lines indicated statistically significant associations, whereas dashed lines indicated non-significant associations. Values on the paths represented unstandardized regression coefficients (*B*). BADL, basic activities of daily living; GDS, geriatric depression scale; MMSE, mini-mental state examination. *n* = 1,611, ^*^p < 0.05.

### 3.5 Sensitivity analyses

Sensitivity analyses generally supported the robustness of the serial mediation findings. After excluding participants with extremely low MMSE scores (MMSE <2), all three specific indirect associations remained statistically significant, and the total indirect association remained significant (*B* = −0.784, 95%CI −0.971 to −0.611, *p* < 0.001). When participants with severe functional dependence (BADL score ≤60) were excluded, the total indirect association remained significant (*B* = −0.524, 95%CI −0.690 to −0.372, *p* < 0.001). The indirect association through depressive symptoms and the serial indirect association through BADL and depressive symptoms also remained significant (*p* < 0.05), whereas the BADL-specific indirect association was attenuated and its 95% bootstrap CI included zero (*B* = −0.085, 95% CI −0.186–0.008, *p =* 0.091). In addition, treating education as a categorical variable using dummy coding yielded results consistent with the primary analysis (*p* < 0.05), with all three specific indirect associations and the total indirect association remaining statistically significant (*p* < 0.05). Detailed results are presented in Table 5.

**Table 5:** Sensitivity analyses of the serial mediation model.

Sensitivity analysis	*n*	Indirect pathway	Unstandardized coefficients (*B*)	95% Boot CI	*p* value
Excluding participants with MMSE <2 (10 excluded)	1,601	Ind 1	−0.308	−0.458 to −0.171	<0.001
		Ind 2	−0.367	−0.476 to −0.271	<0.001
		Ind 3	−0.110	−0.157 to −0.068	<0.001
		Total indirect association	−0.784	−0.971 to −0.611	<0.001
Excluding participants with BADL ≤60 (181 excluded)	1,430	Ind 1	−0.085	−0.186–0.008	0.091
		Ind 2	−0.408	−0.533 to −0.299	<0.001
		Ind 3	−0.031	−0.058 to −0.009	0.013
		Total indirect association	−0.524	−0.690 to −0.372	<0.001
Education modeled categorically using dummy variables	1,611	Ind 1	−0.287	−0.435 to −0.150	<0.001
		Ind 2	−0.401	−0.519 to −0.298	<0.001
		Ind 3	−0.118	−0.168 to −0.075	<0.001
		Total indirect association	−0.806	−0.992 to −0.632	<0.001

Ind1 = Frailty → BADL → MMSE; Ind2 = Frailty → GDS → MMSE; Ind3 = Frailty → BADL → GDS → MMSE. All models were adjusted for age, sex, education, marital status, low-income status, and living arrangement; education was dummy-coded in the third sensitivity analysis. B represents the unstandardized indirect association. Percentile 95% bootstrap CIs were estimated using 5,000 bootstrap samples. Ind, indirect pathway; MMSE, mini-mental state examination; BADL, basic activities of daily living; GDS, geriatric depression scale.

### 3.6 Moderated serial mediation analysis

A moderated serial mediation model was constructed using PROCESS Model 92 (Table 6), and conditional indirect associations at different levels of SSRS for the links are shown in Figure 4. In the model with BADL score as dependent variable, frailty and SSRS were significantly associated with BADL score, alongside a significant frailty–SSRS interaction (*B* = 0.120, 95% CI 0.056–0.185, *p* < 0.001). Moreover, the association between frailty and BADL differed substantially across the three SSRS levels. With increasing SSRS levels, the negative association between frailty and BADL score became weaker, indicating that social support significantly moderated the relationship between frailty and BADL (Figure 4A). In the model with GDS score as the dependent variable, frailty, BADL score and SSRS score were significantly associated with GDS score. However, frailty–SSRS interaction and BADL–SSRS interaction failed to reach statistical significance (all *p* > 0.05). Additionally, the association of frailty or BADL with the GDS score remained comparable across the three SSRS levels (Figures 4B,C). In the model with MMSE score as dependent variable, BADL, GDS, SSRS scores were significantly correlated with MMSE, whereas frailty showed no significant correlation with MMSE. Furthermore, BADL–SSRS interaction and frailty–SSRS interaction were not statistically significant in relation to MMSE. In contrast, GDS–SSRS interaction reached significance (*B* = 0.014, 95% CI 0.005–0.023, *p* = 0.004). Consistently, only the association between GDS and MMSE differed substantially across the three SSRS levels. As SSRS level increased, the negative association between GDS score and MMSE score was attenuated, suggesting that social support moderated the relationship between depression and cognitive function (Figures 4D–F). Taken together, the results indicated social support significantly moderated specific associations between frailty and BADL and between GDS and MMSE.

**Table 6:** The moderated serial mediation model (*n* = 1,611).

Outcome	Variable	Unstandardized coefficients (*B*)	Boot SE	95% Boot CI	*p* value
BADL	Frailty	−6.685	0.444	−7.557 to −5.814	<0.001
	SSRS	0.080	0.033	0.015–0.145	0.016
	Frailty × SSRS	0.120	0.033	0.056–0.185	<0.001
GDS	Frailty	0.737	0.071	0.596–0.877	<0.001
	BADL	−0.029	0.005	−0.040 to −0.019	<0.001
	SSRS	−0.051	0.007	−0.065 to −0.038	<0.001
	Frailty × SSRS	−0.004	0.008	−0.019–0.011	0.577
	BADL × SSRS	0.001	0.001	−0.001–0.001	0.317
MMSE	Frailty	−0.107	0.156	−0.413–0.199	0.492
	BADL	0.036	0.01	0.017–0.056	<0.001
	GDS	−0.426	0.052	−0.528 to −0.323	<0.001
	SSRS	0.056	0.014	0.029–0.083	<0.001
	Frailty × SSRS	−0.012	0.014	−0.039 to 0.015	0.371
	BADL × SSRS	−0.001	0.001	−0.003 to 0.001	0.411
	GDS × SSRS	0.014	0.005	0.005–0.023	0.004

BADL, basic activities of daily living; GDS, geriatric depression scale; MMSE, mini-mental state examination; SSRS, social support rating scale. All models were adjusted for age, sex, education level, marital status, low-income status, and living arrangement.

**Figure 4:**
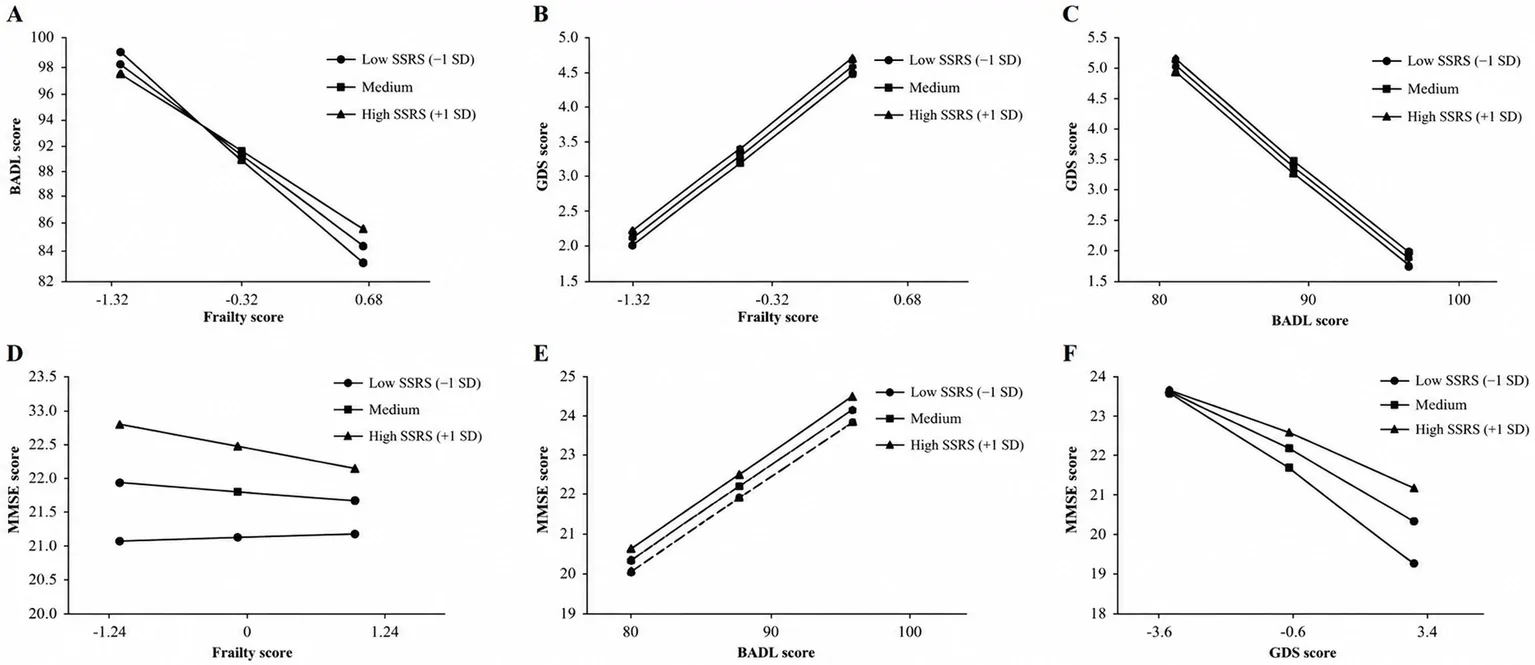
Moderating effects of social support on the associations among frailty, BADL, GDS, and cognitive function. Moderating effects of social support on the associations between: **(A)** frailty with BADL, **(B)** frailty with GDS, **(C)** BADL with GDS, **(D)** frailty with MMSE, **(E)** BADL with MMSE, **(F)** GDS with MMSE. Lines represent the conditional associations at low (≤−1 SD), medium (−1 SD to +1SD), and high (≥+1 SD) levels of SSRS. BADL, basic activities of daily living; SSRS, social support rating scale; GDS, geriatric depression scale; MMSE, mini-mental state examination. *n* = 1,611.

In further analysis, conditional indirect effects at different levels of SSRS for the pathways are shown in Table 7. For the pathway from frailty to MMSE via depression (frailty → GDS → MMSE) and the serial pathway from frailty to MMSE via BADL and then depression (frailty → BADL → GDS → MMSE), the indirect effects at different levels of SSRS were significant. However, for the pathway from frailty to MMSE via BADL alone (frailty → BADL → MMSE), the indirect effects were significant at low and medium levels of SSRS, the effect was nonsignificant at high level of SSRS with 95%CI crossing zero. The moderated mediation analysis showed that the moderated mediation index was significant for the frailty → GDS → MMSE pathway (index = 0.134, 95% CI 0.039–0.227) and was also significant for the serial pathway of frailty → BADL → GDS → MMSE (index = 0.051, 95% CI 0.025–0.078). It was indicated that social support significantly moderated the indirect associations in the pathways from frailty to MMSE via depression alone or via the sequential pathway including BADL and depression.

**Table 7:** Conditional indirect associations at different levels of SSRS (*n* = 1,611).

Pathway	SSRS level	Unstandardized coefficients (*B*)	Boot SE	95% Boot CI
Frailty → GDS → MMSE	Low	−0.465	0.092	−0.662 to −0.304
	Medium	−0.313	0.05	−0.419 to −0.222
	High	−0.179	0.064	−0.317 to −0.064
Frailty → BADL → GDS → MMSE	Low	−0.169	0.042	−0.256 to −0.091
	Medium	−0.084	0.02	−0.126 to −0.049
	High	−0.032	0.016	−0.070 to −0.007
Frailty → BADL → MMSE	Low	−0.369	0.116	−0.603 to −0.152
	Medium	−0.243	0.071	−0.386 to −0.108
	High	−0.144	0.087	−0.323–0.018

Indirect effects were estimated using bootstrap with 5,000 samples. Low, medium, and high represent ≤−1 SD, −1 SD to +1SD, and ≥+1 SD, respectively. The pathways were significant when the 95% CI did not include zero. GDS, geriatric depression scale; MMSE, mini-mental state examination; BADL, basic activities of daily living; SSRS, social support rating scale.

## 4 Discussion

A serial mediation model was used in this study to explore potential pathways underlying the association between frailty and cognitive function in hospitalized older adults. The findings suggested that the relationship between frailty and cognitive function was largely represented by indirect association involving impaired BADL and depressive symptoms within the proposed framework. The pathway involving depressive symptoms showed a relatively larger magnitude, suggesting that psychological factors may represent a relatively prominent component within the statistical framework linking frailty and cognitive function. The serial mediation findings also exhibited good robustness. In addition, social support showed moderating effect in specific pathways, particularly the pathway involving frailty and BADL as well as the pathway involving GDS and cognitive function, underscoring the relevance of psychosocial factors in understanding multidimensional associations among geriatric health domains.

A close association between frailty and cognitive function has been demonstrated in previous studies. Decreased overall cognition and impairments in specific cognitive domains, such as processing speed, have been found in relation to frailty in cross-sectional studies (21). In addition, a cumulative effect has been observed in a longitudinal study, in which persistent frailty or progression toward frailty was associated with an increased risk of subsequent cognitive decline (22). A mechanistic study suggested shared biological pathways between frailty and cognitive decline, including antioxidant capacity, amino acid metabolism, and energy metabolism (23). However, existing research has largely focused on descriptive associations, with limited empirical analyses of specific pathways. Based on this, the potential mediating effect of functional and psychological factors were further examined in the present study. In this study, frailty showed significant indirect association with cognitive impairment involving impaired functional status and depression, reflecting a multi-pathway association pattern. On one hand, the association between frailty and cognitive function involved functional impairment. The decline in physiological reserve reflected by frailty was associated with poorer BADL performance, and functional impairment was also associated with cognitive performance. Previous studies have also shown that frailty was associated with impaired BADL, and functional impairment was closely associated with poorer cognitive function (24, 25). On the other hand, the observed relationship between frailty and cognitive function included an indirect association involving depressive symptom. Furthermore, the indirect association through depressive symptom showed the largest magnitude. Individuals with frailty were more likely to experience negative emotions such as depression, and depression was significantly linked to poorer performance in multiple cognitive domains, including attention, memory, and executive function (26–28). These findings highlight the importance of early identification and management of depressive symptoms for the prevention of cognitive impairment in older adults with frailty, with appropriate psychological support or evidence-based psychological interventions considered when needed. The results of this study further indicate that the psychological pathway may play a more critical role than the functional pathway in the process through which frailty affects cognitive function. Further analysis revealed that the association of frailty with cognitive function involved sequential indirect pathway including functional impairment and depression. Functional impairment was found to restrict activity and social participation, thereby increasing the risk of depression, while depression further exacerbated cognitive decline (11, 29). Previous studies have indicated that functional status plays a mediating role in the relationship between chronic diseases and depression, suggesting a possible linkage between physiological decline and psychological distress (24). In addition, the observed link of frailty with cognitive function was largely represented by indirect association in the current model involving functional status and depressive symptoms. The direct pathway was not statistically significant in this study. Therefore, in clinical practice, attention may be given not only to frailty itself but also to daily living ability and depressive symptoms during comprehensive geriatric assessment, as these factors were associated with cognitive function in the present study.

Social support is generally considered an important protective factor against cognitive decline, yet its specific role in the relationship between frailty and cognitive function remains to be further clarified (30, 31). In this study, social support showed specific moderation for the links between frailty and impaired BADL and between GDS and cognitive function, rather than showing a uniform moderation across the statistical framework. Although the Frailty–SSRS interaction term for cognitive function was significant in the hierarchical regression model, indicating that social support modified the association between frailty and cognitive function before accounting for BADL and GDS, this association was no longer significant after incorporating BADL and GDS into the moderated serial mediation model. This finding suggests that the moderating role of social support may operate through specific functional and psychological pathways rather than directly modifying the overall association between frailty and cognitive function. In addition, as the level of social support increased, the negative association between frailty and impaired BADL became weaker. At the functional level, a previous study has confirmed that social support is closely associated with functional status, with higher levels of social support contributing to the maintenance or improvement of daily living ability and the delay of functional decline (32). Social support has also been shown to modify the association between functional impairment and health outcomes by providing practical assistance, promoting activity participation, and enhancing coping capacity (33). Consistent with this, the present study suggests that social support may moderate the link of frailty with impaired BADL. This finding also suggests the potential value of identifying older adults with limited social support and improving their access to family and community support resources. Social support was also found to moderate the link of depression with cognitive decline. Specifically, the negative association between depressive symptoms and cognitive function was stronger under low levels of social support, while this relationship was attenuated under high levels of social support. At the community level, strengthening social connections and support networks may help reduce social isolation, while health education may encourage older adults to seek help and make better use of available social resources (34, 35). In previous study, social support was reported to modify the association between depressive symptoms and cognitive function through psychosocial mechanisms (36, 37). In contrast, no significant moderating effect of social support was observed in the link of frailty with depression, suggesting a limited moderating association of social support within this link. Instead, social support may show a potential moderating role mainly through modifying the associations between frailty and BADL impairment and between depressive symptoms and cognitive function. In this study, The moderating role of social support in three indirect pathways linking frailty with cognitive function was further analyzed, and significant moderation were observed for the pathways involving frailty, GDS, and MMSE, including the pathway involving GDS alone and the serial pathway involving BADL and GDS. No moderation was shown from frailty to cognitive via BADL alone. The differences among these three pathways may be explained mainly by no significant moderation of social support for the link between BADL and MMSE.

The findings of this study have implications for both clinical practice and public health. The observed associations suggest that cognitive health in older adults is closely linked to multiple dimensions of health, including physical vulnerability, functional status, psychological wellbeing, and social resources. Accordingly, comprehensive approaches that combine frailty management with functional maintenance, psychological support, and enhanced social support may provide a broader framework for supporting cognitive health. Such multidimensional approaches may also contribute to promoting healthy aging among older adults.

Several limitations of this study should be acknowledged. Firstly, the cross-sectional design precludes causal inference regarding the relationships among frailty, functional status, depressive symptoms, social support, and cognitive function. Although the serial mediation model was constructed based on theoretical considerations to explore potential statistical pathways, all variables were assessed at the same time point, and the temporal sequence among frailty, BADL impairment, depressive symptoms, and cognitive function cannot be confirmed. Therefore, the estimated indirect associations and proportion mediated should be interpreted cautiously as statistical associations within the current model rather than evidence of causal mediation. Longitudinal studies are needed to further validate these relationships. Secondly, the study sample was limited to hospitalized older adults from a single-center setting, which may restrict the generalizability of the findings to community-dwelling older populations. Although several demographic and socioeconomic factors were adjusted, residual confounding cannot be completely excluded. Important clinical factors, including multimorbidity burden, nutritional status, medication-related factors, sensory impairment, sleep disturbance, and severity of acute illness, were not fully incorporated into the current models and may have influenced the observed associations. Thirdly, education level was analyzed as an ordered categorical variable based on its inherent hierarchy, which may not fully capture potential differences between educational categories. In addition, MMSE-based cognitive assessment may have potential ceiling effects among participants with relatively preserved cognitive function, which may influence the estimation of associations involving cognitive function. Future studies using alternative coding strategies and more comprehensive cognitive assessments are warranted to further examine the robustness of these findings. Finally, although several associations reached statistical significance, some effect sizes were relatively modest. Given the relatively large sample size, statistical significance alone should not be interpreted as evidence of clinical relevance. Therefore, the findings should be considered together with the magnitude of associations and their potential clinical implications. Despite these limitations, the present study provides evidence that BADL impairment and depressive symptoms may represent important statistical pathways underlying the association between frailty and cognitive function within the proposed framework.

## 5 Conclusion

The relationship between frailty and cognitive function was largely represented by indirect associations involving impaired BADL and depressive symptoms within the proposed framework. Social support moderated specific associations, particularly those between frailty and BADL and between depressive symptoms and cognitive function. These findings support considering functional, psychological, and social factors together when developing comprehensive approaches to maintain cognitive health and promote healthy aging among older adults. From a public health perspective, community-based screening for functional impairment, depressive symptoms, and limited social support, combined with targeted support strategies, may help prevent cognitive impairment in frail older adults.

## Data Availability

The raw data supporting the conclusions of this article will be made available by the authors, without undue reservation.
