# Supplementary material for: Association between frailty and cognitive function in hospitalized older adults: a moderated serial mediation analysis involving functional status, depression, and social support
Source: Front Public Health. 2026 Sep 28;14:1895486. doi: 10.3389/fpubh.2026.1895486 (PMC13655232; doi:10.3389/fpubh.2026.1895486)
Supplement: Supplementary file 1 [file Table_1.docx]

**Supplementary Table S1.** Comparison of available baseline characteristics between included and excluded participants

| **Variable** | **Category** | **Excluded** | | **Included** | | **t / χ²** | **P value** |
| --- | --- | --- | --- | --- | --- | --- | --- |
|  |  | **N** | **Mean ± SD / n (%)** | **N** | **Mean ± SD / n (%)** |  |  |
| Age, years | — | 173 | 74.46 ± 8.67 | 1,611 | 75.67 ± 8.50 | −1.746 | 0.082 |
| Sex |  | 173 |  | 1,611 |  |  |  |
|  | Male |  | 80 (46.2) |  | 787 (48.9) | 0.426 | 0.514 |
|  | Female |  | 93 (53.8) |  | 824 (51.1) |  |  |
| Education level |  | 154 |  | 1611 |  |  |  |
|  | Illiterate |  | 49 (31.8) |  | 511 (31.7) | 1.128 | 0.89 |
|  | Primary school |  | 70 (45.5) |  | 756 (46.9) |  |  |
|  | Junior high school |  | 20 (13.0) |  | 171 (10.6) |  |  |
|  | Senior high school |  | 6 (3.9) |  | 80 (5.0) |  |  |
|  | College or above |  | 9 (5.8) |  | 93 (5.8) |  |  |
| Marital status |  | 173 |  | 1,611 |  |  |  |
|  | Married |  | 148 (85.5) |  | 1,323 (82.1) | 1.268 | 0.26 |
|  | Widowed/divorced/unmarried |  | 25 (14.5) |  | 288 (17.9) |  |  |
| Low-income |  | 159 |  | 1,611 |  |  |  |
| status | No |  | 145 (91.2) |  | 1,396 (86.7) | 2.649 | 0.104 |
|  | Yes |  | 14 (8.8) |  | 215 (13.3) |  |  |
| Living alone |  | 149 |  | 1,611 |  |  |  |
|  | No |  | 140 (94.0) |  | 1,523 (94.5) | 0.087 | 0.767 |
|  | Yes |  | 9 (6.0) |  | 88 (5.5) |  |  |
|  |  |  |  |  |  |  |  |
| Smoking status |  | 154 |  | 1,611 |  |  |  |
|  | Never |  | 106 (68.8) |  | 1,196 (74.2) | 2.179 | 0.336 |
|  | Former |  | 29 (18.8) |  | 244 (15.1) |  |  |
|  | Current |  | 19 (12.3) |  | 171 (10.6) |  |  |
| Alcohol consumption |  | 147 |  | 1,611 |  |  |  |
|  | Never |  | 117 (79.6) |  | 1,352 (83.9) | 4.762 | 0.092 |
|  | Former |  | 22 (15.0) |  | 152 (9.4) |  |  |
|  | Current |  | 8 (5.4) |  | 107 (6.6) |  |  |
| BADL score | — | 159 | 90.28 ± 16.65 | 1,611 | 89.02 ± 19.01 | 0.808 | 0.419 |
| GDS score | — | 157 | 3.91 ± 3.35 | 1,611 | 3.60 ± 3.30 | 1.104 | 0.271 |
| SSRS score | — | 171 | 35.69 ± 11.27 | 1,611 | 35.66 ± 12.02 | 0.037 | 0.970 |
| Frailty score | — | 162 | 1.14 ± 1.14 | 1,611 | 1.32 ± 1.24 | −1.775 | 0.076 |
| MMSE score | — | 124 | 22.59 ± 6.30 | 1,611 | 21.68 ± 6.57 | 1.550 | 0.123 |

**Note:** BADL: basic activities of daily living, GDS: geriatric depression scale, SSRS: social support rating scale, MMSE: mini-mental state examination.
